# Mass-producible disposable needle-type ion-selective electrodes for plant research†

**DOI:** 10.1039/c9ra05477d

**Published:** 2019-09-25

**Authors:** Md. Abunasar Miah, Yusei Nakagawa, Ryo Tanimoto, Rina Shinjo, Motohiko Kondo, Hiroaki Suzuki

**Affiliations:** Department of Biotechnology and Genetic Engineering, Faculty of Science, Noakhali Science and Technology University Noakhali-3814 Bangladesh; Graduate School of Bioagricultural Sciences, Nagoya University Furo-cho, Chikusa-ku Nagoya Aichi 464-8601 Japan; Graduate School of Pure and Applied Sciences, University of Tsukuba 1-1-1 Tennodai Tsukuba Ibaraki 305-8573 Japan; Itochu Sugar Co., Ltd. Tamatsu-ura 3 Hekinan Aichi 447-8506 Japan; Aichi Agricultural Research Center Susogaeto 11, Inabu Toyota Aichi 441-2513 Japan

## Abstract

Easily mass-producible needle-type Na^+^ and K^+^ ion-selective electrodes (ISEs) were developed for the direct and indirect measurement of Na^+^ and K^+^ ion concentrations in live plants. A polyimide strip with a silver layer on one side and Ag/AgCl formed at one end was used to construct two types of ISEs. For the type I ISE, an electrolyte layer was formed on the layer of silver and Ag/AgCl, which was then covered with a protecting layer. Subsequently, an ion-selective membrane (ISM) was formed at the truncated end with Ag/AgCl. For the type II ISE, a syringe needle was used as a container and an ISM was formed at the sharp end. Then, the polyimide strip with Ag/AgCl at one end was inserted and an electrolyte solution was injected to complete the ISE. Reference electrodes (REs) with similar structures were fabricated by forming a liquid junction instead of the ISM. The electrode responses and the relationship between the ISE potential and the Na^+^/K^+^ ion concentration agreed with those predicted by the Nernst equation. The Na^+^ and K^+^ ion concentrations in different parts of the rice plant (*Oryza sativa* L.) were measured using the Na^+^ and K^+^ ISEs, respectively. The results obtained using these devices agreed well with those obtained using inductively coupled plasma atomic emission spectrometry, thus confirming the practical applicability of the developed ISEs.

## Abstract 2

Easily mass-producible needle-type ion-selective electrodes were developed for the direct and indirect measurement of ion concentrations in live plants.

## Graphical abstract

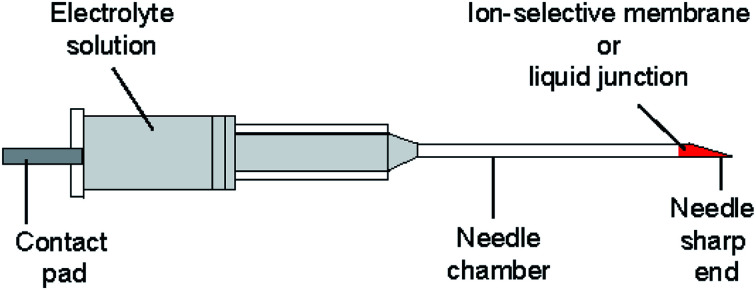

## Introduction

Rice is an important staple food in many countries all over the world. However, its production yield is decreasing significantly owing to problems related to soil salinity that make cultivable lands unfertile, which makes it challenging to increase the cropping intensity of rice to a sustainable level.^1–3^ To ensure that production keeps pace with population growth, the selection of salt-tolerant rice with a high production yield is critical. Although salt-tolerant rice can be identified by traditional plant breeding,^4^ such phenotypic field screening is time-consuming, labor-intensive, and costly.^5^

To overcome the issues associated with phenotypic field screening, the concentrations of ions have been used as indicators to identify salt-tolerant plants. In most cases, the Na^+^ and K^+^ ion concentrations in tissues have been used as indicators for plant breeders.^6,7^ In most species, Na^+^ ions are known to accumulate to toxic levels when the roots are exposed to high concentrations of NaCl. By contrast, excess K^+^ ions are known to suppress increases in the Na^+^ ion concentration.^8^ The amounts of Na^+^ and K^+^ ions in tissues such as leaves and roots have been measured using techniques such as atomic absorption spectroscopy,^9^ flame photometry,^10^ mass spectrometry,^11^ and inductively coupled plasma mass spectrometry.^12^ In addition, at the subcellular level, techniques such as X-ray microanalysis have been used.^13^ These methods are accurate and reliable, even for samples with low ion concentrations. However, these techniques require expensive instruments and cumbersome sample preparation procedures including grinding, ashing, extraction, and filtration using dangerous reagents, such as sulfuric acid, nitric acid, hydrogen chloride, or hydrogen peroxide.^14^ Therefore, rapid and easy techniques with simple sample preparation procedures are needed to measure ion concentrations in live plant tissues.

To meet these criteria, the ion-selective electrode (ISE) is an attractive device.^15,16^ ISEs allow ion concentrations to be determined based on the measurement of a potential difference generated at an ion-selective membrane (ISM). To measure ion concentrations in very small regions or small sample volumes, ISEs made with glass capillaries have been used, particularly for physiological research.^17,18^ However, special care is needed in handling these fragile structures. In addition, such ISEs are not suitable for mass production. To solve these problems, we applied techniques used for the microfabrication of semiconductor chips. Hundreds of thin polyimide strips with Ag/AgCl electrodes were easily mass-produced and then used to construct ISEs of two types. We also used this technique to fabricate needle-type reference electrodes (REs) that can be used in combination with the ISEs. Our technique not only realizes inexpensive disposable ISEs but also enables measurements in sample solutions with very small volumes or directly in plants. These ISEs were successfully applied to measure Na^+^ and K^+^ ion concentrations in rice plants.

## Experimental

### Reagents and materials

The materials and reagents used for the fabrication and characterization of the devices were purchased from the following commercial sources: a polyimide sheet (thickness: 130 μm) from JMT Corporation (Osaka, Japan); a positive photoresist (S-1818G) from Dow Chemical (Midland, MI, USA); bis(12-crown-4) and 2-nitrophenyloctyl ether (NPOE) from Dojindo (Kumamoto, Japan); valinomycin, dioctyl sebacate (DOS), tetrahydrofuran (THF), polyvinylpyrrolidone (PVP), polyvinyl chloride (PVC), potassium chloride (KCl), and sodium chloride (NaCl) from Wako Pure Chemical Industries (Osaka, Japan); and sodium tetraphenylborate, poly(2-hydroxyethyl methacrylate) (poly(HEMA)), and potassium tetrakis(4-chlorophenyl)borate (KTpClPB) from Sigma Aldrich (Buchs, Switzerland). Syringe needles (inner diameter: 0.9 mm, outer diameter: 1.3 mm) were purchased from Terumo (Tokyo, Japan). Solutions were prepared with Milli-Q water (Millipore, Tokyo, Japan).

### Preparation of solutions for ISMs

To form the Na^+^ ISM, 88.8 mg (29.6 wt%) of PVC was dissolved in 3 mL of THF. Then, 200 mg (66.6 wt%) of NPOE, 10 mg (3.3 wt%) of bis(12-crown-4), and 1.5 mg (0.5 wt%) of sodium tetraphenylborate were added. The mixture was thoroughly stirred to mix completely.^19^ The solution for the K^+^ ISM was prepared using 6.25 mg (2.5 wt%) of ligand (valinomycin), 1.0 mg (0.4 wt%) of lipophilic additive (KTpClPB), 159.5 mg (63.8 wt%) of plasticizer (DOS), and 83.25 mg (33.3 wt%) of PVC.^20^ The components were dissolved in 2.5 mL of THF. Finally, the mixture was stirred until the membrane components were completely mixed.

### Structure and fabrication of type I ISEs

**Fig. 1:**
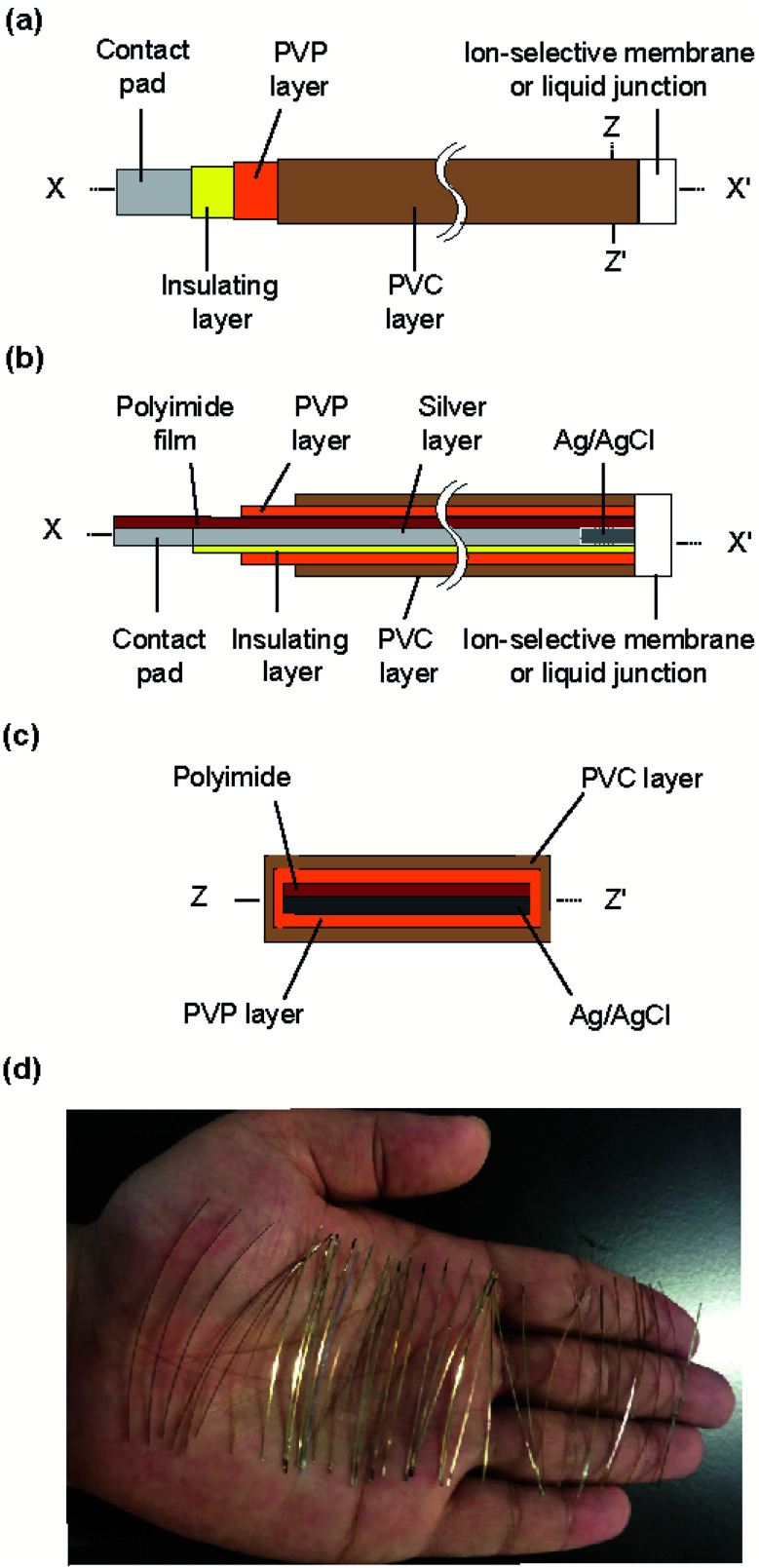
Structure of the type I ISE and RE: (a) top view, (b) cross section along the line *x*–*x*′, and (c) cross section along the *z*–*z*′ line. (d) Photograph of the polyimide strips with a silver layer.

The structure of the type I ISE and RE is shown in Fig. 1. This ISE consisted of a silver electrode with Ag/AgCl at one end and an electrolyte layer covered with a PVC coating that served as a container. Furthermore, an ISM was formed at one end.

**Fig. 2:**
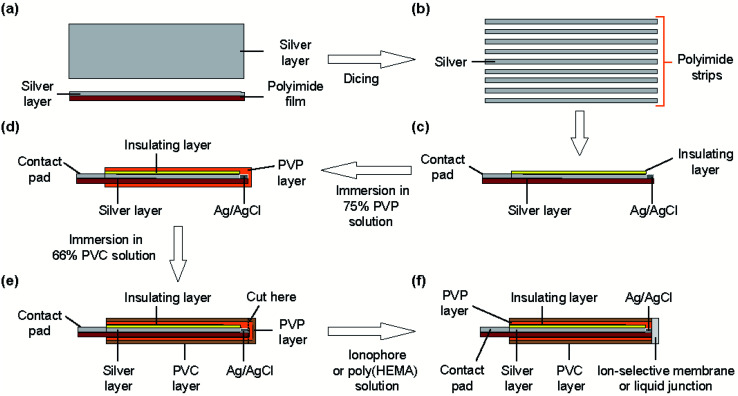
Fabrication process for the type I ISE and RE. (a) Top and cross-sectional views of a polyimide sheet with a silver layer. (b) Top view of diced polyimide strips (0.5 mm × 50 mm) with a silver layer. (c) Cross section of a strip with a silver layer and an insulating layer of the positive photoresist. AgCl was formed at one end of the silver layer. (d) Formation of the electrolyte layer containing PVP. (e) Formation of the PVC layer that acted as a reservoir. (f) Formation of an ISM or liquid junction at the truncated end to obtain a type I ISE or RE, respectively.

Fig. 2 shows the fabrication steps for the type I ISE. First, to improve the adhesion of silver to the substrate, the surface of a polyimide sheet (80 mm × 60 mm) was roughened by sandblasting and then cleaned by sonication. The sonication step was repeated three times, using fresh acetone every time. A 600 nm thick silver layer was sputter-deposited on one side of the sheet. A positive photoresist was coated on the silver layer for protection. Next, the polyimide sheet with a silver layer was cut into thin strips of 0.5 mm × 50 mm using a dicing machine, and the positive photoresist was removed using acetone. The silver layer on the strip was again coated with the positive photoresist, but both ends were left exposed. One exposed end (∼8 mm) was used to grow AgCl and the other (∼10 mm) was used as a contact pad. AgCl was grown by immersing the exposed end of silver in a 100 mM NaCl solution and applying a constant current (50 nA) for 10 min using a platinum counter electrode and a galvanostat (HA-151, Hokuto Denko, Japan).^21^

To form the electrolyte layer for type I Na^+^ ISEs, a 75 wt% PVP solution containing 100 mM NaCl was prepared. The strip with Ag/AgCl was immersed in the PVP solution and then removed immediately. The PVP layer was allowed to dry for 30 min. A 66 wt% PVC solution was then prepared in THF. The electrode with the PVP layer was immersed in the PVC solution, leaving part (∼3 mm) of the PVP layer exposed, and then the PVC layer was allowed to dry for 10 min. The end of the coated strip (∼2 mm) was then truncated using scissors. To form the ISM for type I Na^+^ ISEs, the truncated end was placed in contact with the PVC solution containing the Na^+^ ionophore and removed immediately. This step was repeated twice. The ISM was then allowed to dry for at least 24 h before the fabricated ISE was used for measurements. The type I K^+^ ISEs were fabricated in the same manner, except 100 mM KCl was used to prepare the PVP solution and the solution containing the K^+^ ionophore was used to form the ISM. The thickness of the ISM was approximately 90 μm.

### Structure and fabrication of type II ISEs

**Fig. 3:**
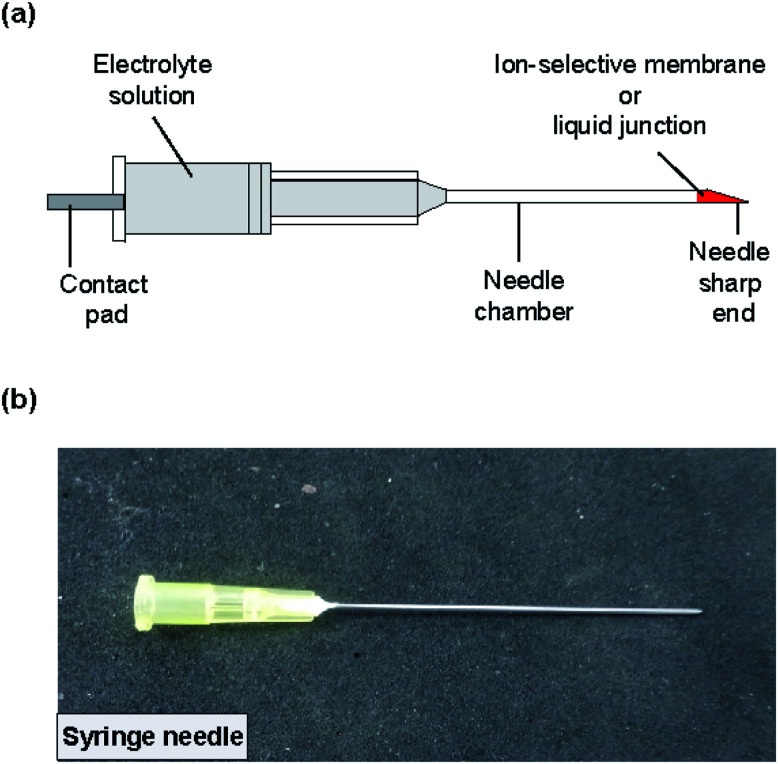
(a) Structure of the type II ISE and RE. The ISM or liquid junction was formed at the sharp end of the needle. (b) Photograph of the completed ISE.

The structure of the type II ISE and RE is shown in Fig. 3. A syringe needle was used as a container for a Ag/AgCl electrode and an electrolyte solution, and an ISM was formed at the sharp end of the needle. To form the ISM, the syringe needle was cleaned with ethanol for 10 min using a sonicator and then dried on a hotplate for 20 min at 120 °C. The sharp end of the needle was placed in contact with the same ISM solution as used for the type I ISE, and then the solvent was allowed to evaporate for 24 h at room temperature. The thickness of the ISM was approximately 90 μm. Next, the same polyimide strip with Ag/AgCl as used for the type I ISE and the electrolyte solution (100 mM NaCl for the Na^+^ ISE or 100 mM KCl for the K^+^ ISE) were introduced into the syringe needle. Fig. S1† shows how the electrolyte solution was introduced into the syringe needle. Briefly, the syringe needle with the polyimide strip with Ag/AgCl was vertically inserted into the electrolyte solution in a centrifuge tube with the ISM orientated towards the bottom. Then, the tube was placed in a vacuum chamber, and the solution was introduced into the needle by evacuation.

### Structure and fabrication of type I and II REs

The type I and II REs had the same structures and were fabricated using the same procedures as the type I and II ISEs, respectively, except that the composition of the PVP solution was different and a liquid junction was used instead of an ISM. Although many attempts have been made to realize miniature REs,^22–24^ the use of the dried electrolyte layer or the syringe needle facilitated the formation of liquid junctions, as in the case of ISMs.

For the type I RE, the electrolyte layer was formed by dip coating using a solution containing 75 wt% PVP and 3.0 M KCl. The end of the polyimide strip with Ag/AgCl and the electrolyte layer was truncated using scissors. To form the liquid junction, 32.5 mg of poly(HEMA) was dissolved in 500 μL ethanol, and the solution was thoroughly stirred to mix well. The truncated end of the polyimide strip was placed in contact with this solution to seal the open end with a liquid junction. This step was repeated five times and then the membrane was allowed to dry for 24 h.

To fabricate the type II RE, a liquid junction was formed at the sharp end of the syringe needle before introducing the electrolyte solution. The sharp end of the syringe needle was placed in contact with the same poly(HEMA) solution as used for the type I RE. This step was repeated five times and the membrane was allowed to dry for 24 h. After the liquid junction was formed, a polyimide strip with Ag/AgCl was placed inside the syringe needle and a 3.0 M KCl solution was introduced into the interior of the type II RE by evacuation (Fig. S1†). In the KCl solution, a drop of AgNO_3_ solution was added to suppress the dissolution of AgCl.

### Locations of measurements in rice plants

**Fig. 4:**
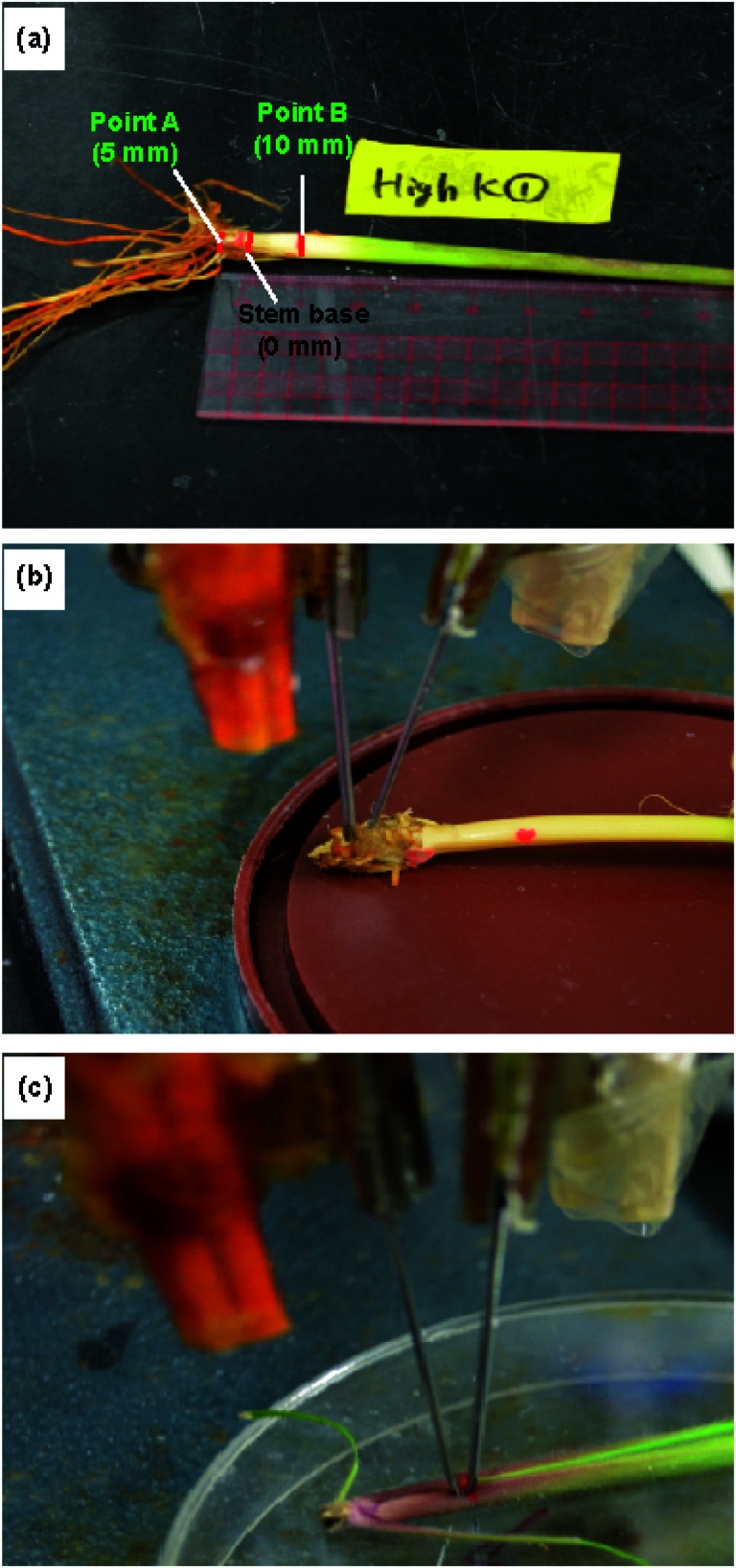
Experimental setup for measurements using the type II ISEs: (a) locations of the stem at which the ion concentrations were measured, and the ISE and RE inserted at (b) point A and (c) point B.

The ion concentrations were measured at the roots, stem base, stem, and leaves. Furthermore, we measured the ion concentrations at two different points on the stem. The stem base, corresponding to the uppermost part of an elongated internode, was selected as the origin (0 mm). “Point A” was located 5 mm below the stem base and “point B” 10 mm above the stem base (Fig. 4 and S2†).

### Growth of rice plants and preparation of sample powders

Rice plants (cv. Tachisugata) were grown in a pot of 1/5000 a (1 a = 100 m^2^) under different Na^+^ and K^+^ ion levels. Uniform basal application of 0.5 g N per pot, 0.2 g P_2_O_5_ per pot, and 0.2 g K_2_O per pot was carried out for each pot. For high-concentration Na^+^ and K^+^ treatments, 10 mL of 500 mM NaCl or KCl solution, respectively, was added into each pot near the booting stage. The plants were harvested at maturity and separated into different plant parts. The samples were ground to a fine powder after drying at 80 °C.

### Preparation of extract solutions for ICP-AES analysis

To prepare extract solutions for inductively coupled plasma atomic emission spectrometry (ICP-AES) measurements, a standard method^25^ was used with slight modifications. First, 20 ± 0.2 mg of the dried powder sample was placed in a disposable centrifuge tube, then 300 μL of nitric acid was added and the solution was kept overnight. Subsequently, the tube was heated in an oven for 2 h at 105 °C to digest the sample powder completely. Next, the tube was allowed to cool for 30 min or longer, 10 mL of Milli-Q water was added, and the solution was mixed well using a vortex mixer. An aliquot of the solution was diluted with Milli-Q water and mixed using the vortex mixer. Finally, the sample solution was filtered through filter paper (pore diameter: 0.45 μm).

### Preparation of extract solutions for type I ISE analysis

For the measurements using the type I ISEs, extract solutions were prepared using the root, stem base, stem, and green and dead leaves of rice plants (Fig. S2†). First, 10 ± 0.5 mg of the dried powder was placed in a disposable centrifuge tube, Milli-Q water was added to obtain a volume of 1.0 mL, and the solution was mixed thoroughly. Finally, the sample solution was centrifuged for 2 min at 15 000 rpm to remove any debris.

### Measurement of sodium and potassium concentrations in dry powdered samples

To determine the concentrations of sodium and potassium in dry powdered rice samples, the concentrations of Na^+^ and K^+^ ions in the extract solutions were first measured by ICP-AES (IRIS AP, Nippon Jarrell Ash Co., Ltd., Tokyo, Japan). Using the solution concentrations, the concentrations of sodium and potassium on a dry weight basis in the samples, *A*, with respect to the dry weight of the rice sample were calculated based on the following equation.*A* (mg kg^−1^) = *a* (mg L^−1^) × *b* (L kg^−1^)Here, *a* is the concentration of the extract solution and *b* is the ratio between the volume of the total extract solution and the dry weight of the sample.

The measurements using the type I ISEs and the subsequent calculations were conducted in the same manner. First, calibration plots for the ISEs were obtained using Na^+^ or K^+^ standard solutions. Then, the sensitive area of the ISEs and the liquid junction of the corresponding REs were immersed in the extract solutions. The potential of the ISE with respect to the RE was measured using an electrometer (AutoLab PGSTAT12, Eco Chemie, Utrecht, Netherlands). The concentrations of sodium and potassium in the dry rice samples were then calculated as in the case of ICP-AES.

### Preparation of rice samples for type II ISE analysis

For the measurements of intact plants using the type II ISEs, the ratoons of rice plant (cv. Nipponbare) grown in a field were transplanted to a pot of 1/10 000 a and grown under low- and high-concentration Na^+^ and K^+^ conditions for 54 days. For the low-concentration Na^+^/K^+^ treatments, 2.5 mL of 200 mM NaCl/KCl solutions were added, whereas 12.5 mL of 200 mM NaCl/KCl solutions were added for the high-concentration Na^+^/K^+^ treatments. On the 10th day after the Na^+^/K^+^ treatment, the plant was removed carefully from the soil and immediately used for measurement after cleaning with water and wiping with tissue paper. The locations of the measurements and the experimental setup are shown in Fig. 4.

### Measurements of Na^+^ and K^+^ ion concentrations in rice plants

Prior to the measurements using the type I Na^+^/K^+^ ISEs and REs, the internal dried electrolyte layer was wetted by immersion of the electrodes in 100 mM NaCl or KCl (for ISEs) and 3 M KCl (for REs) for 5–10 min. Prior to use, the ISEs were immersed in a 100 mM NaCl or KCl solution for 30 min for preconditioning. The responses of the type I Na^+^/K^+^ ISEs were examined using NaCl and KCl standard solutions, respectively, or the prepared sample solutions. For each measurement, an ISE and RE of the same type were used. For comparison, a commercial RE (2060A, Horiba, Kyoto, Japan) was also used to construct calibration plots. The ISE and RE were immersed in a solution and the potential of the ISE with respect to the RE was measured using an electrometer (AutoLab PGSTAT12, Eco Chemie, Utrecht, Netherlands). After one measurement, the ISM of the ISE and the liquid junction of the RE were rinsed with distilled water, dried with tissue paper, and then immersed in the next solution. The same steps were repeated for all standard solutions.

The measurements using the type II ISEs were conducted in the same manner. The ISEs were immersed in a 100 mM NaCl or KCl solution for 30 min for preconditioning prior to use. Then, the ISE and corresponding RE were inserted directly into point A or point B of the stem of an intact live rice plant (Fig. 4). The potential of the ISE was measured with respect to the RE, as described for the type I ISEs. The same ISE and RE were used for measurements until the ISM or liquid junction broke. The performance of the ISE and RE was checked in a standard solution after every 5th insertion into a plant. For statistical analysis, different rice plants were used, with each rice plant discarded after the measurement. The Na^+^ and K^+^ ion concentrations were calculated based on calibration plots constructed using NaCl and KCl standard solutions, respectively. The internal electrolyte solutions of the type II ISEs and REs were replenished with fresh solution prior to each measurement. All the experiments with type I and type II ISEs were conducted at 25 °C.

## Results and discussion

### Performance of the REs and ISEs

**Fig. 5:**
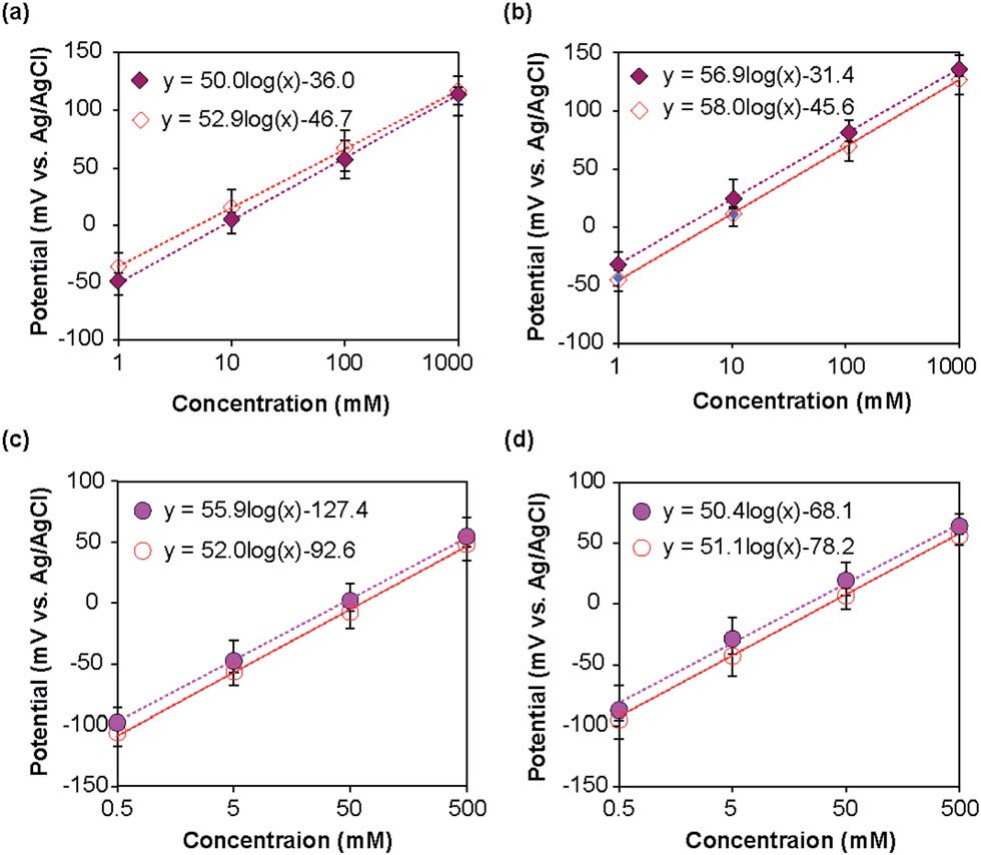
Calibration plots for the ISEs: (a) type I Na^+^ ISE, (b) type II Na^+^ ISE, (c) type I K^+^ ISE, and (d) type II K^+^ ISE. The open symbols represent measurements relative to the corresponding type I or type II RE, whereas the closed symbols represent measurements relative to the commercial RE.

The potentials of the ISEs were measured with respect to the corresponding REs. For the type I and type II Na^+^ ISEs, the measured potential changed with the change in Na^+^ ion concentration of the standard NaCl solutions, as shown in Fig. 5a and b. As anticipated based on the Nernst equation, a linear relationship was observed for both types of ISEs in the examined concentration range, and the potential shifted in the positive direction with the increase in Na^+^ ion concentration. For the type I Na^+^ ISE, the slopes of the obtained plots with respect to the type I and commercial REs were +52.9 and +50.0 mV per decade, respectively. The slopes of the plots for the type II Na^+^ ISE with respect to the type II and commercial REs were +58.0 and +56.9 mV per decade, respectively. The measured slopes were slightly smaller than those anticipated from the Nernst equation (59.2 mV per decade at 25 °C).

In the same manner, the type I and II K^+^ ISEs were characterized using KCl standard solutions with respect to the corresponding REs and a commercial Ag/AgCl electrode. Linear relationships were observed for both types of ISEs in the examined concentration range (Fig. 5c and d), and the potential shifted in the positive direction with the increase in K^+^ ion concentration. The slopes of the calibration plots for the type I K^+^ ISE with respect to the type I and commercial REs were +52.0 and +55.9 mV per decade, respectively. The slopes of the plots for the type II K^+^ ISE with respect to the type II and commercial REs were +51.1 and +50.4 mV per decade, respectively. These values were also slightly smaller than those expected based on the Nernst equation.

### Measurements of Na^+^ and K^+^ ion concentrations in rice plants

**Fig. 6:**
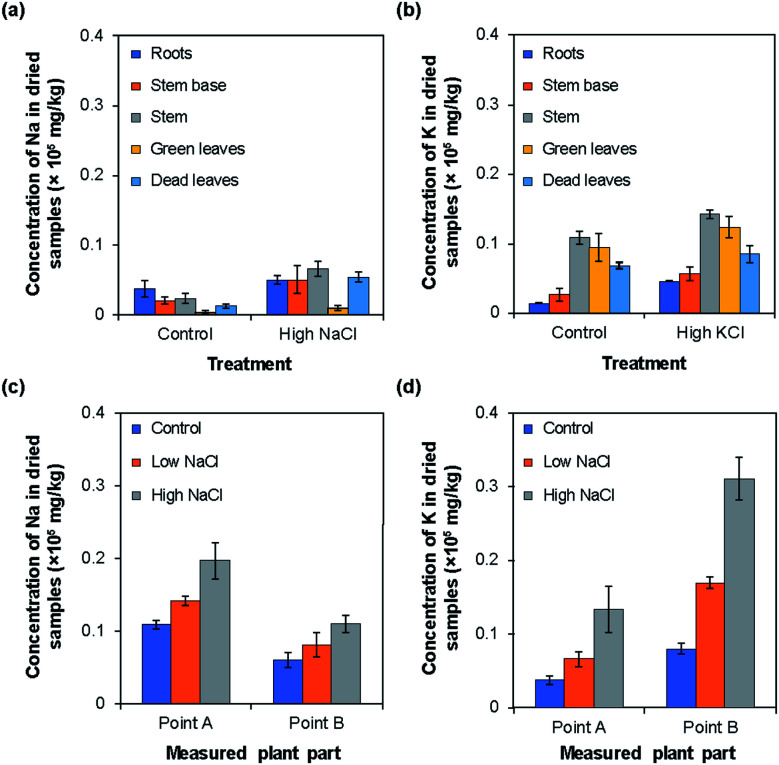
Concentrations of sodium and potassium in the dried parts of rice plants measured using type I ISEs. (a) Concentration of sodium in the dried parts of rice plants (cv. Tachisugata) grown normally (control) and under high-concentration Na^+^ treatment. (b) Concentrations of potassium in the dried parts of rice plants (cv. Tachisugata) grown normally (control) and under high-concentration K^+^ treatment. (c) Concentration of sodium in the dried parts of rice plants (cv. Nipponbare) at points A and B under treatments with different NaCl concentrations. (d) Concentration of potassium in the dried parts of rice plants (cv. Nipponbare) at points A and B under treatments with different KCl concentrations.

To measure Na^+^ or K^+^ ion concentrations using the type I and II ISEs, rice plants were grown under normal (control), low, and high NaCl or KCl concentrations. The results obtained using the type I ISEs are shown in Fig. 6. Fig. 6a and b show the influence of growth under high Na^+^/K^+^ ion concentrations, with the results obtained under normal conditions shown for comparison. When the rice was grown under a high Na^+^ concentration, the Na^+^ ion concentrations were higher than the control values in all the examined tissues. In addition, the Na^+^ ion concentration was higher in the stem than in the other plant parts. When the rice was grown under a high K^+^ ion concentration, the tendency was similar to that observed for Na^+^ ions. Overall, the K^+^ ion concentrations in the plant parts grown under a high K^+^ concentration were higher than the control values. In addition, the K^+^ ion concentrations were higher in the stem and green leaves than in the other plant parts.

Fig. 6c and d show the Na^+^/K^+^ ion concentrations at points A and B of rice grown under low and high Na^+^/K^+^ concentrations, with the results obtained under normal conditions shown for comparison. Overall, the Na^+^ ion concentration was higher at point A than at point B. Moreover, the Na^+^ ion concentration at both points increased with the increase in Na^+^ ion concentration applied during growth (Fig. 6c). By contrast, the K^+^ ion concentration was higher at point B than at point A. However, as in the case of Na^+^ ions, the K^+^ ion concentration increased with the increase in the K^+^ ion concentration applied during growth. In addition, in all parts of the rice plant, the change with increasing ion concentration was larger for K^+^ ions than for Na^+^ ions. Similar tendencies have been reported for the Na^+^ and K^+^ ion contents of plants measured by atomic absorption spectroscopy,^9^ flame photometry,^10^ mass spectrometry,^11^ and inductively coupled plasma mass spectrometry.^12^Table 1 compares the results obtained using the type I Na^+^ and K^+^ ISEs with those obtained by the ICP-AES method. Overall, the values obtained using the two methods agreed well. It should be noted that the sample preparation procedure for the type I ISE measurements is much simpler and faster than that for ICP-AES measurements, although the ground parts of rice plants were used for both methods.

**Table 1:** Comparison of the concentrations of sodium and potassium in the dried parts of rice plants (cv. Tachisugata) determined using type I ISEs and ICP-AES

Measured plant part	Treatment	Type I Na^+^ ISE × 10^5^ (mg kg^−1^)	ICP-AES × 10^5^ (mg kg^−1^)	Treatment	Type I K^+^ ISE × 10^5^ (mg kg^−1^)	ICP-AES × 10^5^ (mg kg^−1^)
Roots	Control	0.037	0.025	Control	0.013	0.016
	High Na^+^	0.050	0.032	High K^+^	0.046	0.129
Stem base	Control	0.020	0.030	Control	0.027	0.022
	High Na^+^	0.050	0.044	High K^+^	0.056	0.024
Stem	Control	0.023	0.042	Control	0.108	0.100
	High Na^+^	0.066	0.042	High K^+^	0.142	0.113
Green leaves	Control	0.003	0.004	Control	0.094	0.108
	High Na^+^	0.009	0.005	High K^+^	0.123	0.124
Dead leaves	Control	0.012	0.007	Control	0.068	0.072
	High Na^+^	0.054	0.032	High K^+^	0.085	0.091

**Fig. 7:**
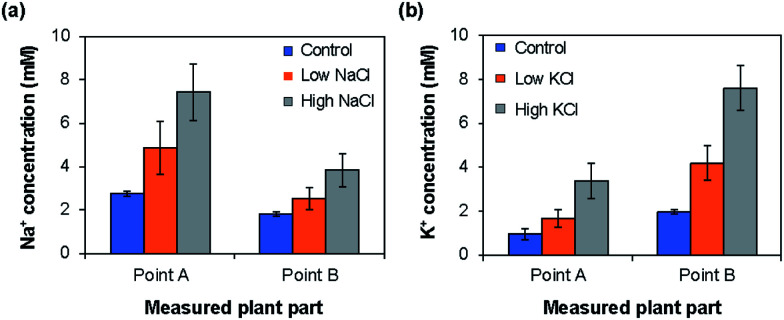
Distributions of (a) Na^+^ and (b) K^+^ ions at points A and B in live rice plants (cv. Nipponbare) measured using type II ISEs for rice grown normally (control) and under low- and high-concentration (a) Na^+^ and (b) K^+^ treatments.

Depending on the case, it may be better to measure the ion concentrations by inserting the ISEs directly into the plant part of interest. To this end, we used the type II ISEs, which were inserted into points A and B of 64 days old seedlings of rice plants (cv. Nipponbare) grown under low and high Na^+^/K^+^ ion concentrations. As shown in Fig. 7, the Na^+^ ion concentration was higher at point A than at point B, whereas the K^+^ ion concentration was higher at point B than at point A. Furthermore, the Na^+^ and K^+^ ion concentrations in these parts increased with the increase in the salt concentration in the root during growth. This tendency agrees with the results obtained using the type I ISEs (Fig. 6c and d). In addition, the Na^+^ ion concentration was higher than the K^+^ ion concentration at point A, whereas the opposite tendency was observed at point B, indicating K^+^, an essential element for plants, and Na^+^ exhibit different dynamics. Overall, the distributions of the ions measured using the type II ISEs were similar to those obtained using the type I ISEs and ICP-AES. These results also agree with those obtained previously by another group.^26^ Thus, the type II ISEs can be used for the direct measurement of ion concentrations and their localization in live intact rice plants.

## Conclusions

Needle-type ISEs were fabricated for the measurement of ion concentrations in plants. Because they are very thin, the fabricated ISEs can be used to measure ion concentrations in sample solutions with very small volumes or directly in the plant. In addition, these inexpensive and disposable ISEs can be mass-produced easily. The type I ISEs were fabricated by stacking a Ag/AgCl electrode, an electrolyte layer, a coating layer, and an ISM. By contrast, the type II ISEs were fabricated by inserting a strip with a Ag/AgCl electrode into a syringe needle with an ISM at the end. These ISEs can be stored in a dried state and then activated prior to use by introducing an electrolyte solution. REs of the same structure were fabricated in the same manner by forming a liquid junction using poly(HEMA). The REs used in combination with the ISEs provided stable potentials corresponding to those expected for a Ag/AgCl electrode for a sufficiently long time to allow measurements. The ion concentration values obtained using the type I ISEs agrees well with those obtained by the conventional ICP-AES method. Furthermore, as the type II ISE could be inserted into the hard parts of a plant, they allowed the ion concentrations to be measured directly. Using this approach, ISEs for other ions can also be realized. The developed ISEs will be a useful tool for the efficient screening of salt-tolerant plants.

In using the ISEs for real sample analyses, there are two points to be considered. One is the initial drift of the ISE potential after placing the ISM in contact with the internal and external solutions. The other is storage and user-friendliness. Because thin-film Ag/AgCl electrodes are easily damaged,^27^ the ISEs should be stored in a dry state until use. Furthermore, long preconditioning times are not beneficial and users should be able to use the ISEs immediately after preparation. To promote widespread use of the ISEs, further optimization will be needed to address these issues.

## Conflicts of interest

There are no conflicts of interest to declare.

## Supplementary Material

RA-009-C9RA05477D-s001
