# Supplementary material for: Mass-producible disposable needle-type ion-selective electrodes for plant research
Source: RSC Adv. 2019 Sep 25;9(52):30309–16. doi: 10.1039/c9ra05477d (PMC9072120; doi:10.1039/c9ra05477d)
Supplement: RA-009-C9RA05477D-s001 [file RA-009-C9RA05477D-s001.pdf]

Electronic Supplementary Material (ESI) for RSC Advances.
This journal is © The Royal Society of Chemistry 2019

## Supplementary Material (ESI) for RSC Advances

# Mass-producible disposable needle-type ion-selective electrodes for plant research

Md. Abunasar Miah,[a, c] Yusei Nakagawa,[b] Ryo Tanimoto,[b] Rina Shinjo,[b] Motohiko Kondo[b] and Hiroaki Suzuki*

[a] Department of Biotechnology and Genetic Engineering, School of Life Sciences, Noakhali Science and Technology University, Noakhali-3814, Bangladesh.

[b] Graduate School of Bioagricultural Sciences, Nagoya University, Furo-cho, Chikusa-ku, Nagoya, Aichi, 464-8601, Japan.

[c] Graduate School of Pure and Applied Sciences, University of Tsukuba, 1-1-1 Tennodai, 305-8573, Tsukuba, Japan.

Contents

1. Introduction of the electrolyte solution into the syringe needle chamber.
2. Figure S2

### 1. Introduction of the electrolyte solution into the syringe needle

Fig. S1 shows how the electrolyte solution was introduced into the syringe needle. In brief, the polyimide stripe with the Ag/AgCl electrode was inserted into the syringe needle. Then, the syringe needle was vertically inserted into the centrifuge tube with the ISM orientated towards the bottom. Next, the centrifuge tube was placed in a vacuum chamber, and the electrolyte solution was introduced into the needle by vacuum evacuation.

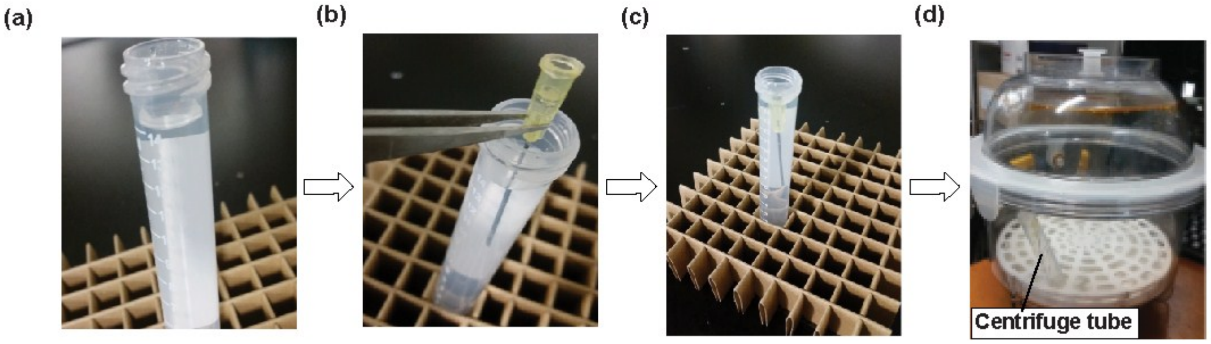


**Fig. S1** Introduction of the electrolyte solution into the syringe needle. (a) A centrifuge tube is filled with the electrolyte solution. (b) The syringe needle with the ISM is inserted vertically into the solution. (c) The syringe needle is completely immersed in the solution. (d) The tube is placed in a desiccator and the solution is introduced into the syringe needle by evacuation.

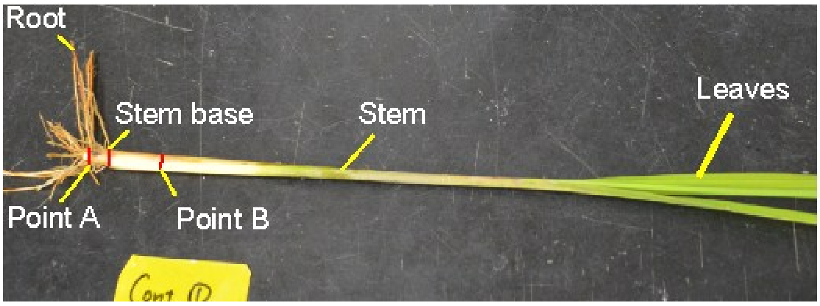


**Fig. S2** Rice plant parts and locations used for measurements the type I and II ISEs.
